# The microbiome of lower respiratory tract and tumor tissue in lung cancer manifested as radiological ground-glass opacity

**DOI:** 10.3389/fbioe.2022.892613

**Published:** 2022-08-25

**Authors:** Zhigang Wu, Jie Tang, Runzhou Zhuang, Di Meng, Lichen Zhang, Chen Gu, Xiao Teng, Ziyue Zhu, Jiacong Liu, Jinghua Pang, Jian Hu, Xiayi Lv

**Affiliations:** ^1^ Department of Thoracic Surgery, The First Affiliated Hospital, School of Medicine, Zhejiang University, Hangzhou, China; ^2^ Department of Thoracic Surgery, Fenghua People’s Hospital, Ningbo, China

**Keywords:** microbiome, 16S rRNA sequencing, ground-glass opacity, lung cancer, biomarker

## Abstract

Recent studies have confirmed the existence of microbiota in the lungs. The relationship between lung ground-glass opacity (GGO) and microbiota in the lung microenvironment is not clear. In this study, we investigated the microbial composition and diversity in bronchoalveolar lavage fluid (BALF) of diseased lung segments and paired contralateral healthy lung segments from 11 GGO patients. Furthermore, lung GGO and paired normal tissues of 26 GGO patients were explored whether there are microbial characteristics related to GGO. Compared with the control group, the community richness of GGO tissue and BALF of GGO lung segment (α-diversity) and overall microbiome difference (β-diversity) had no significant difference. The microbiome composition of BALF of GGO segments is distinct from that of paired healthy lung segments [genus (*Rothia*), order (*Lachnospiraceae*), family (*Lachnospiraceae*), genus (*Lachnospiraceae_NK4A136_group*, *Faecalibacterium*), and species (*Faecalibacterium prausnitzii*, *Bacteroides uniforms*)]. GGO tissue and adjacent lung tissue had more significant differences at the levels of class, order, family, genus, and species level, and most of them are enriched in normal lung tissue. The area under the curve (AUC) using 10 genera-based biomarkers to predict GGO was 91.05% (95% CI: 81.93–100%). In conclusion, this study demonstrates there are significant differences in the lower respiratory tract and lung microbiome between GGO and the non-malignant control group through the BALF and lung tissues. Furthermore, some potential bacterial biomarkers showed good performance to predict GGO.

## Introduction

Low-dose computed tomography (LDCT) is widely used as the main method of lung cancer screening project wordwide, and an increasing number of lung ground-glass opacity (GGO) are found (Aberle et al., 2011). Pulmonary nodules are round shadows with a diameter of less than 3 cm in chest CT images. Among pulmonary nodules, GGO are defined as lesions with higher opacity than normal lung tissue, but lower than the consolidated bronchovascular edge (MacMahon et al., 2017). Although GGO is a non-specific radiologic manifestation, persistent and long-term stable GGO is generally considered to be malignant and still considered to be an inert and progressed slowly subtype of lung adenocarcinoma (Chang et al., 2013). Therefore, the causes of GGO have attracted the attention of clinicians and researchers. GGO usually does not have driver gene mutations, which usually occurred in lung adenocarcinomas, such as EGFR and ALK (Ren et al., 2019a). Benign lesions including infectious diseases such as COVID-19 can also be radiographed as GGO. Pathologically, GGO can be caused by interstitial thickening with inflammation, edema, fibrosis, and tumor proliferation (Fan et al., 2012). Meanwhile, epidemiological studies have also suggested that there is a close relationship between chronic infection, inflammation, and lung cancer (Gomes et al., 2014). Therefore, the microbiome may play an important role in the occurrence of early adenocarcinoma characterized by GGO.

At present, the most research on microbiome and diseases is the correlation between intestinal microbiome and some metabolic diseases or gastrointestinal cancer. However, with the development of high-throughput next-generation sequencing (NGS), the entire spectrum of the human microbiome has been surveyed; recent studies show that in addition to intestinal microbiome, symbiotic microbiome also exists in other locations of the human body. In the past, it was considered that the lung is a sterile space, but recent studies have suggested that the lower respiratory tract is also full of various bacterial communities, which is very important in maintaining the stability of the internal environment and can cause respiratory diseases such as asthma, COPD, and lung cancer (Hilty et al., 2010; Mao et al., 2018; Maddi et al., 2019; Ramírez-Labrada et al., 2020). Compared with gastrointestinal cancer, there are few studies on the correlation between microbiome and lung cancer. Epidemiological studies have shown the correlation between repeated exposure to antibiotics and increased risk of lung cancer (Boursi et al., 2015), but the effect of lung microbiome on lung cancer is still unknown.

Some studies have confirmed that there are some unique microbiota in BALF, sputum, saliva, or lung tissue of patients with lung cancer (Lee et al., 2016; Yu et al., 2016; Cameron et al., 2017; Liu et al., 2018a; Tsay et al., 2018; Peters et al., 2019; Zhang et al., 2019; Mao et al., 2020), and these studies have not only found similar but also contradictory microbiota prevalent in patients with lung cancer. However, previous studies have mostly compared the microbial composition of bronchoalveolar lavage fluid from lung cancer patients and healthy people, or tumor tissue and normal lung tissue from typical lung cancer patients. Few studies explored the microbiome composition of GGO lesions. In this study, we screened BALF from 11 patients with GGO, fresh frozen GGO lung tissue, and paired adjacent lung tissue from 26 patients. Furthermore, the microbial diversity of lower respiratory tract and lung tissue of patients with GGO and the identified characteristic microbiome were revealed, which also provides a new idea for the occurrence and treatment of GGO.

## Materials and methods

### Patient enrollment and sample collection

The enrolled patients were from patients who underwent radical resection of lung cancer in the First Affiliated Hospital of Medical College of Zhejiang University from September 2019 to September 2021. Twenty six lung tumor specimens and paired normal lung tissues were collected, and bronchoalveolar lavage fluid of 11 diseased lung segments and paired contralateral healthy lung segments were collected. The included patients did not use antibiotics or adjuvant therapy 3 months before operation; HRCT showed pulmonary ground-glass nodules; lung cancer was diagnosed by pathology; and no previous history of other cancers. Bronchoalveolar lavage fluid (15ml) from the diseased lung segment and the contralateral healthy lung segment was centrifuged and enriched and put into liquid nitrogen. The tumor tissue was removed under sterile conditions and immediately put into liquid nitrogen, and then transferred to the −80° refrigerator for preservation until DNA extraction. While collecting tumor tissue, collect adjacent normal lung tissue more than 5 cm away from tumor lesion to avoid local influence of tumor. At the same time, a blank control tube is designed to run through the whole sample collection process, and then delivered in dry ice container to Novogene Inc. (Beijing, China) for 16S rRNA gene sequencing.

### DNA extraction

Total genome DNA from BALF and lung tissue samples was extracted using the CTAB method. DNA concentration and purity were monitored on 1% agarose gels. According to the concentration, DNA was diluted to 1 ng/μl using sterile water.

### 16S rRNA gene sequencing

16S rRNA genes of distinct regions (16S V4/16S V3/16S V3-V4/16S V4-V5) were amplified using specific primer with the barcode. All PCR reactions were carried out with 15 µl of Phusion® High-Fidelity PCR Master Mix (New England Biolabs). Mix same volume of 1X loading buffer (contained SYBR Green) with PCR products and operate electrophoresis on 2% agarose gel for detection. PCR products were mixed in equidensity ratios. Then, mixture PCR products were purified with the Qiagen Gel Extraction Kit (Qiagen, Germany). Sequencing libraries were generated using TruSeq® DNA PCR-Free Sample Preparation Kit (Illumina, United States), following manufacturer’s recommendations, and index codes were added. The library quality was assessed on the Qubit@ 2.0 Fluorometer (Thermo Scientific) and Agilent Bioanalyzer 2100 system. At last, the library was sequenced on an Illumina NovaSeq platform and 250 bp paired-end reads were generated.

### Data analysis

Sequences with ≥97% similarity were assigned to the same OTUs. Representative sequence for each OTU was screened for further annotation. OTUs abundance information was normalized using a standard of the sequence number, corresponding to the sample with the least sequences. Subsequent analysis of alpha diversity and beta diversity were all performed based on this output normalized data. Alpha diversity is applied in analyzing complexity of species diversity for a sample through two indices, including Shannon and Simpson. All this indices in our samples were calculated with QIIME (Version 1.7.0) and displayed with R software (Version 2.15.3). Beta diversity analysis was used to evaluate differences of samples in species complexity; beta diversity on both weighted and unweighted UniFrac was calculated by QIIME software (Version 1.9.1). PERMANOVA was used to test the statistical significance of diversity differences between groups. The linear discriminant analysis (LDA) score by LEfSe (LDA effect size) was used to estimate taxa features with significant differential abundance. The random forest model was performed to estimate the importance of each differential genus and test predictive power based on the area under the receiver operating characteristic curve (ROC).

## Results

### Patient characteristics

A total of 37 patients with ground-glass nodules were included in the study. All patients had no other lung comorbidities. They were confirmed as lung cancer by pathology. Among all patients, 11 patients underwent bronchoscopy, and bronchoalveolar lavage fluid was collected before operation, and 26 patients underwent radical resection of lung cancer and collected surgical specimens. The clinical characteristics of the two groups of patients are shown in Table 1.

**TABLE 1:** Baseline clinical characteristics of the study cohort.

Clinical characteristic	BALF group (*n* = 11)	GGO group (*n* = 26)
Age (years; mean ± SD)	51.81 ± 9.06	51.54 ± 9.84
Sex (female)	9 (81.82%)	20 (76.92%)
Smoking (yes)	1 (9.09%)	5 (23.08%)
Multiple (yes)	2 (18.18%)	7 (26.92%)
Lesion location		
Upper left	3 (27.27%)	6 (23.08%)
Lower left	2 (18.18%)	4 (15.38%)
Upper right	3 (27.27%)	10 (38.46)
Middle-lower right	3 (27.27%)	6 (23.08)
Surgery type		
Wedge resection	6 (54.55%)	14 (53.85%)
Segmentectomy	5 (45.45%)	9 (34.62%)
Lobectomy	0	3 (11.53%)
Tumor diameter (cm; mean ± SD)	0.82 ± 0.15	0.91 ± 0.23
Histology		
AIS	0	2 (7.69%)
MIA	7 (63.63%)	16 (61.54%)
IAC	4 (36.37%)	8 (30.77%)

### Lower respiratory tract microbiota in lung segment with ground-glass opacity and contralateral normal lung segment

**FIGURE 1:**
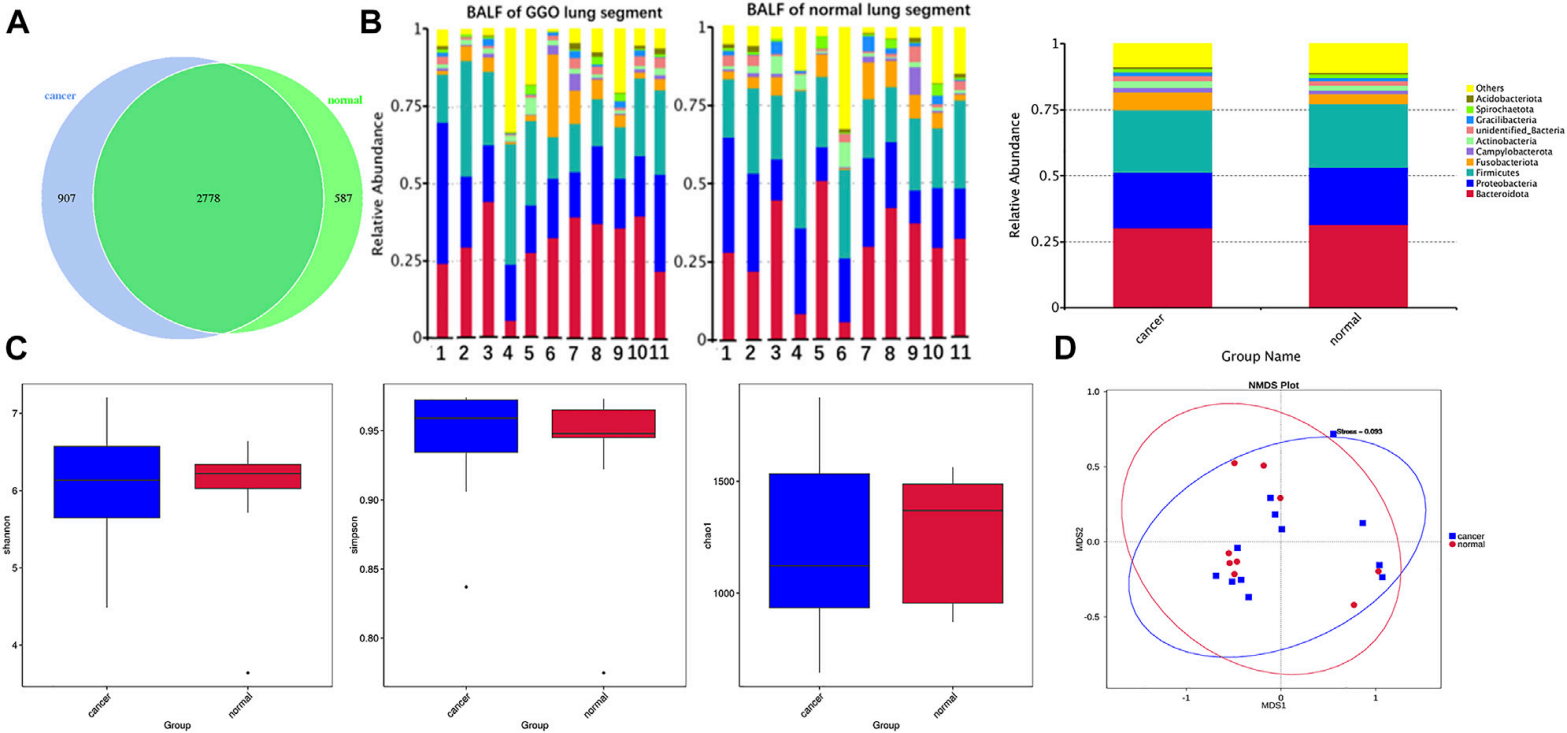
Microbial composition and diversity in BALF of the lung segment with GGO and contralateral normal lung segment. **(A)** Operational taxonomic units (OTUs) between GGO and normal BALF groups. **(B)** Bar plot presents the relative abundance of microbial phyla in each sample and groups. **(C)** Shannon, Simpson, and Chao1 index of GGO and normal BALF groups (*p* > 0.05). **(D)** Non-metric multidimensional scaling (NMDS) plot visualizes the overall microbiome dissimilarity measured by Bray–Curtis dissimilarities.

Splicing and quality control were performed to obtain effective tags for subsequent analysis through the Illumina NovaSeq sequencing platform. An average of 87,624 tags was measured per sample, and an average of 70,471 valid data was obtained after quality control. The effective rate of quality control was 80%. The operational taxonomic units (OTUs) were clustered with 97% identity, and a total of 4,272 OTUs were obtained with 3,685 OTUs in BALF of a lung segment with GGO and 3,365 in BALF of a contralateral normal lung segment (Figure 1A), and the sequence of OTUs was annotated finally. The richness and diversity of microbial community (α-diversity) in BALF samples of the lung segment with GGO and contralateral normal lung segment were measured by Chao1 index, Shannon index, and Simpson index had no significant difference (Figure 1C). PERMANOVA analysis based on the Bray–Curtis dissimilarity (Figure 1D), unweighted, and weighted UniFrac boxplot (Supplementary Figure S1) revealed that there were no significant differences in the overall microbiota (β-diversity) between two groups of BALF.

**FIGURE 2:**
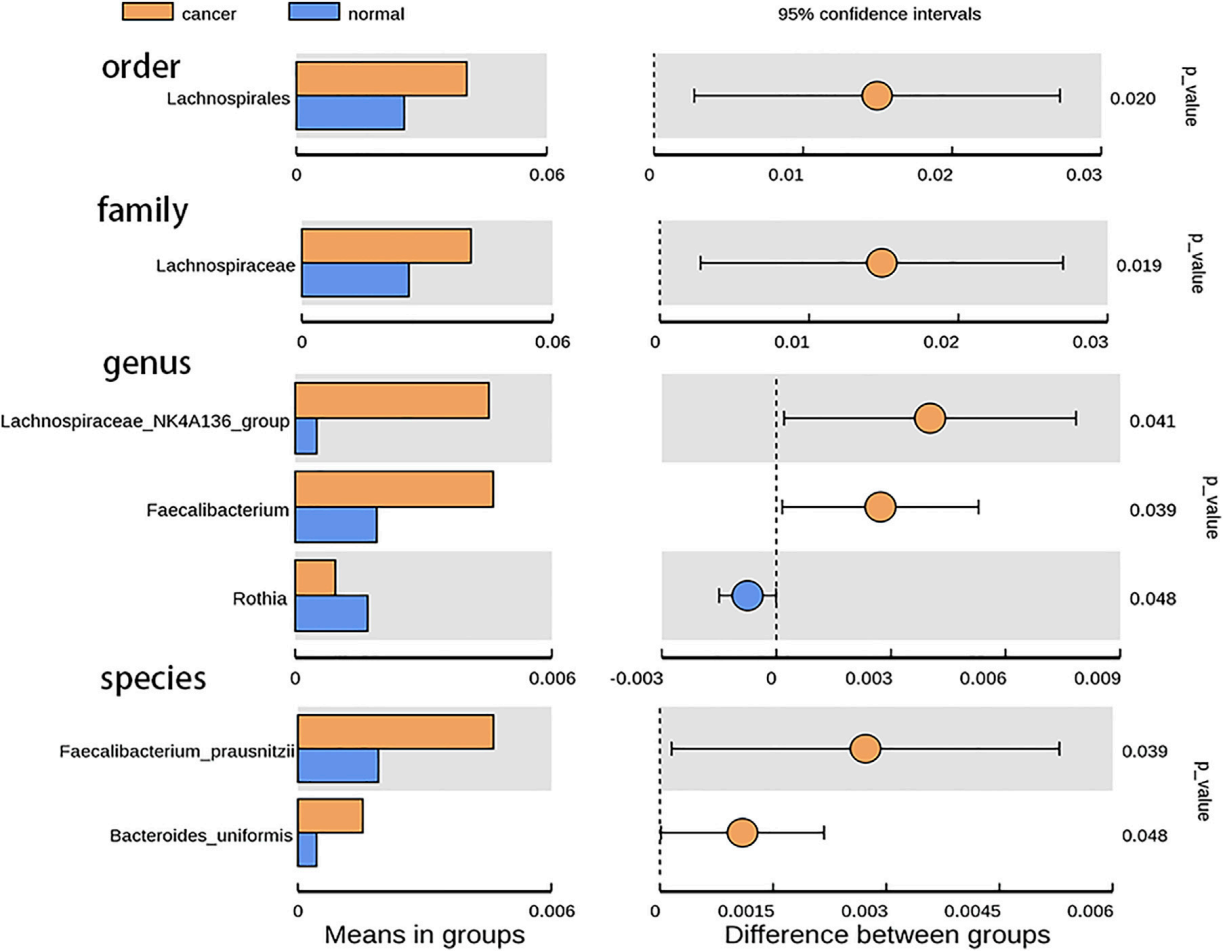
Bar plot presents the microbiota with significant differential relative abundance on the phylum, class, order, family, genus, and species levels between BALF of lung segment with GGO and contralateral normal lung segment.

According to the relative abundance of the microbiota in the BALF samples of the two groups, classification and analysis were based on the phylum, class, order, family, genus, and species levels (Supplementary Figure S2). At the phylum level, the most abundant compositions were *Bacteroidota*, *Proteobacteria*, *Firmicutes*, *Fusobacteriota*, and *Actinobacteria* in both BALF of the lung segment with GGO and contralateral normal lung segment (Figure 1B). However, there was no significant difference in the phylum level of the main flora between the two groups. In addition, at the genus level, *Rothia* is more enriched in BALF of the normal lung segment (*p* < 0.05) (Figure 2). Furthermore, the relative abundance of microbiota at order (*Lachnospiraceae*), family (*Lachnospiraceae*), genus (*Lachnospiraceae_NK4A136_group, Faecalibacterium*), and species (*Faecalibacterium prausnitzii*, *Bacteroides uniforms*) level is increased significantly in BALF of the lung segment with GGO (*p* < 0.05) (Figure 2).

### The composition and diversity of lung microbiota in lung ground-glass opacity and paired adjacent normal tissue

**FIGURE 3:**
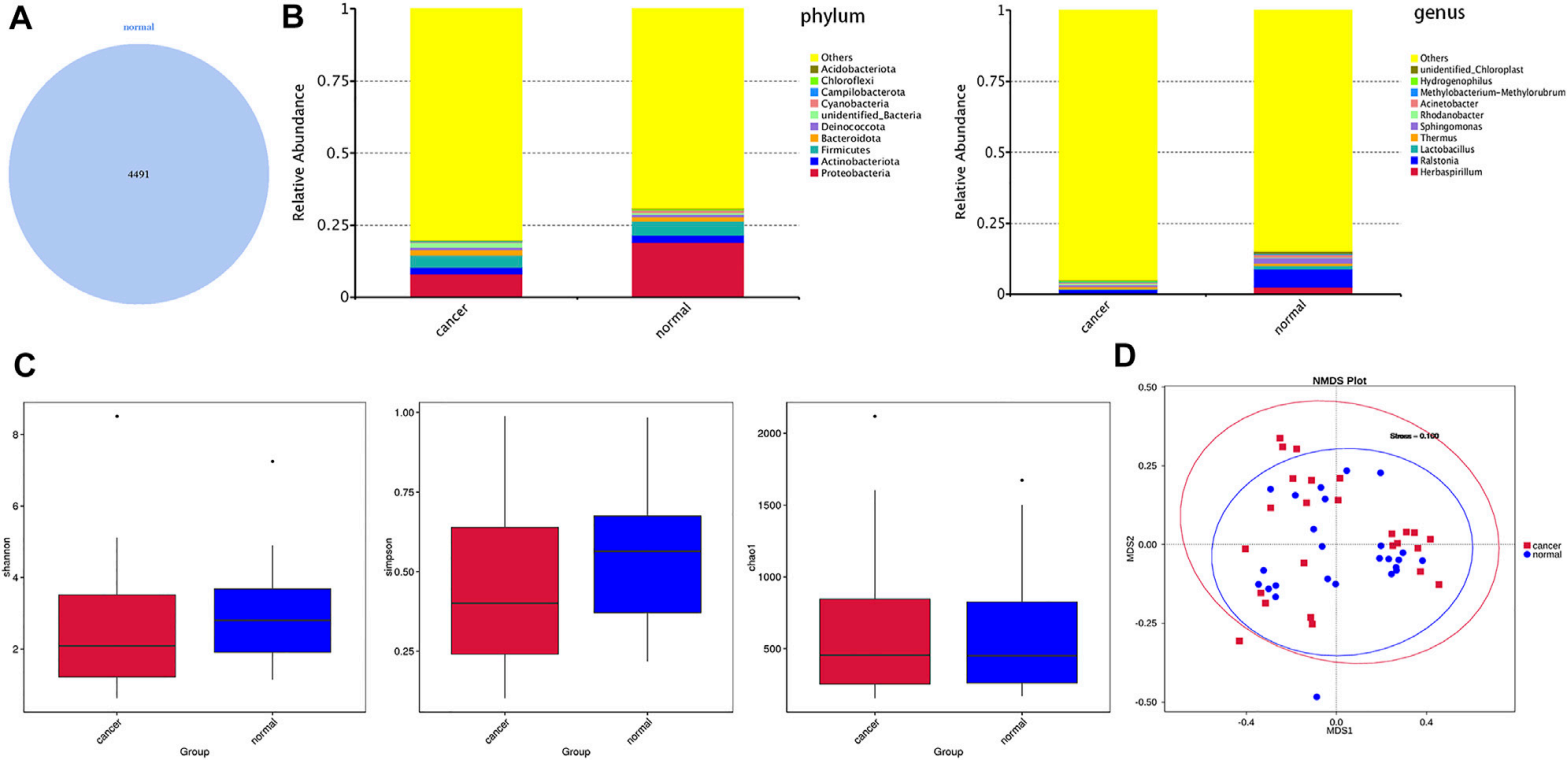
Microbial composition and diversity in lung GGO and paired adjacent normal tissues. **(A)** Operational taxonomic units (OTUs) between GGO and normal tissues. **(B)** Bar plot presents the relative abundance of microbial phyla and genera in GGO and normal lung tissues. **(C)** Shannon, Simpson, and Chao1 index of GGO and normal groups (*p* > 0.05). **(D)** Non-metric multidimensional scaling (NMDS) plot visualizes the overall microbiome dissimilarity measured by Bray–Curtis dissimilarities.

**FIGURE 4:**
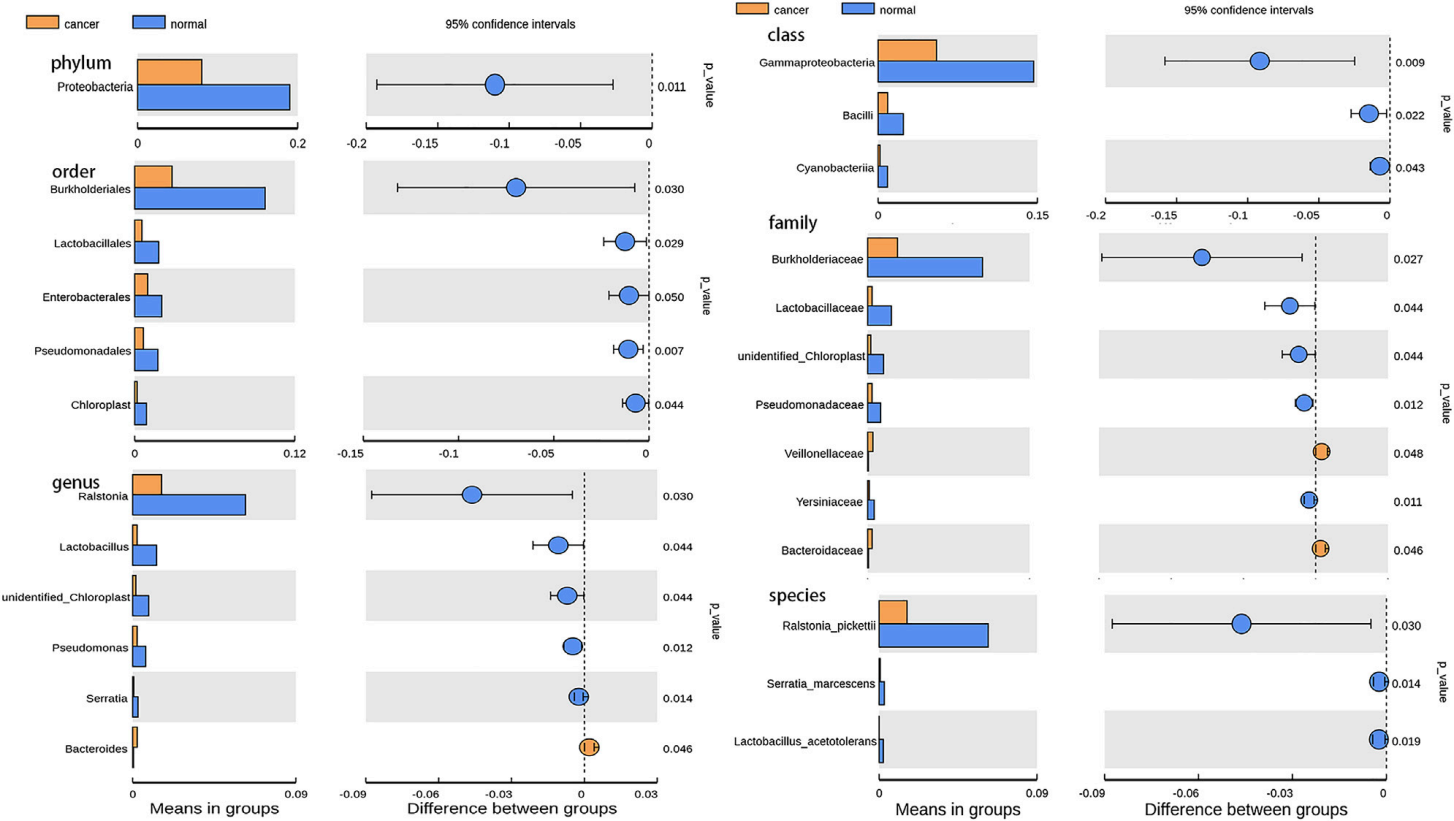
Bar plot presents the microbiota with significant differential relative abundance on the phylum, class, order, family, genus, and species levels between lung GGO and paired adjacent normal tissues.

Based on the Illumina NovaSeq sequencing platform, lung tissue samples were sequenced and analyzed similar to BALF samples to obtain OTUs for subsequent analysis. GGO tumor tissue and paired adjacent normal lung tissue had the same total of 4,491 OTUs, which is much more than BALF samples (Figure 3A). The main phyla in the microbiome of GGO tissues and adjacent non-malignant tissues include *Proteobacteria*, *Actinobacteria*, *Firmicutes*, and *Bacteroidetes*, and the most abundant genera were *Ralstonia*, *Herbaspirillum*, and *Sphingomonas* (Figure 3B). In addition, there are significant differences in *Proteobacteria* between tumor tissues and normal tissues in main phyla (Figure 4). However, α-diversity which was estimated by Chao1 index, Shannon index, and Simpson index and PERMANOVA analysis (β-diversity) based on Bray–Curtis dissimilarity (Figures 3C,D), unweighted, and weighted UniFrac boxplot were of no significant difference between GGO tissues and adjacent tissues, which was the same as BALF samples. GGO tissue and adjacent lung tissue had significant differences in the composition of flora at the levels of class, order, family, genus, and species as shown in Figure 4, and interestingly most of them are enriched in normal lung tissue.

### Potential biomarkers for ground-glass opacity based on bacterial taxa feature

**FIGURE 5:**
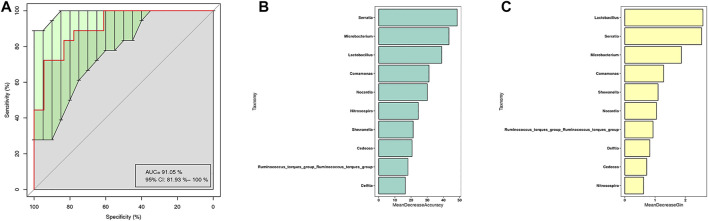
Random forest model based on bacterial taxa feature to distinguish GGO tissue and adjacent normal lung tissue. **(A)** Receiver operating characteristic (ROC) curves with the 10 significant differential genera (AUC = 91.05%) to predict GGO and paired adjacent normal tissue. **(B)** Mean decrease accuracy measures the degree of reduction in the accuracy of random forest prediction by changing the value of a variable into a random number. The higher the value, the greater the importance of the variable. **(C)** Mean decrease Gini calculates the influence of each variable on the heterogeneity of observations at each node of the classification tree through Gini index, so as to compare the importance of variables. The higher the value, the greater is the importance of the variable.

The receiver operating characteristic (ROC) analysis was performed to evaluate the diagnostic ability of potential biomarkers in GGO based on the 10 different genera of GGO tissue and adjacent normal lung tissue, and the calculated area under the curve (AUC) represented the diagnostic performance of each biomarker. The AUC produced by 10 difference genera was 91.05% (95% CI: 81.93–100%) (Figure 5A), which were proven to be effective in distinguishing GGO and paired adjacent normal tissue. The importance ranking of the 10 difference genera included in the random forest analysis was demonstrated by mean decrease accuracy (Figure 5B) and mean decrease Gini (Figure 5C).

## Discussion

The past view was that the lungs of healthy people were sterile. However, with the development of high-throughput NGS, several studies recently confirmed the existence of microbiota in healthy lungs (Dickson et al., 2016), which overturned the past cognition, and the lung microbiota was associated with human health and disease status and played an important role in cancer progressing (Maddi et al., 2019). In this study, we confirmed that there was obvious microbiota in the BALF and tissue samples of patients with GGO, which provides the research direction and clue of tumor microbiome for the occurrence of GGO.

Because the bacteria content of healthy lung is very small, the external pollution in the process of sample collection and experiment has a great impact on the results (Salter et al., 2014). It is very important to set up a negative control in the study of lung microbiome. In our study, we collected BALF and lung tissue removed by aseptic surgery. In order to avoid pollution in the process of sampling and DNA extraction, we designed a negative control respectively. The results showed that the DNA concentration of the negative control was very low and could not be amplified by PCR, which ruled out the influence of external pollution on the results.

In the current study of lung microbiome, most of them are studied through BALF and brush samples. Because bronchoscopy needs to enter the lower respiratory tract through the upper respiratory tract, there is a risk of sample contamination. However, studies have shown that the microbiota in BALF obtained by bronchoscopy is not affected (Dickson et al., 2016). Therefore, BALF is feasible as a research method of lower respiratory tract microbiome.

Some studies have confirmed that in chronic lung diseases, the flora structure of lower respiratory tract will change, such as COPD (Mammen and Sethi, 2016) and bronchiectasis (Budden et al., 2019). Differences in the overall structure of lung microbiome composition between lung cancer and non-malignant diseases were observed, which was consistent with the results of Liu et al. (2018b); Tsay et al. (2018), indicating that there were significant differences in the composition of pulmonary microbial communities between the two groups. In our study, we found that there were differences in the overall structure of microbial communities between the two groups by NMDS analysis, which suggested that there were significant differences in the microbial composition of the lower respiratory tract between the lung segment with GGO and the contralateral normal lung segment. The results of a diversity analysis showed that there was no significant difference in the richness and diversity of microbiota between the BALF samples of the diseased and normal lung segment, which was similar with the conclusions of Jin’s study on BALF microbiome in patients with lung cancer and healthy patients (Jin et al., 2019), indicated that the microbiome composition of the lower respiratory tract is very similar to that of the upper respiratory tract, and the oral flora may be the main source of the respiratory tract flora (Dickson and Huffnagle, 2015). In our study, the four main phyla of BALF are *Bacteroidota*, *Proteobacteria*, *Firmicutes*, *Fusobacteriota*, and *Actinobacteria*, which are consistent with the results of other studies on the composition of microbiota in BALF at the phylum level and commonly found in the oral cavity (Jin et al., 2019; Cheng et al., 2020). Cheng et al. (2020) found phylum TM7 and six genera were enriched in the lung cancer group compared with the control group by comparing BALF samples from patients with lung cancer (*n* = 32) and patients with benign lung disease (*n* = 22). However, the same microbiome differences were not found in our study, but we found that there was greater abundance of family *Lachnospiraceae* in BALF of GGO patients. Some studies have found that richness of family *Lachnospiraceae* is related to the low survival rate of lung cancer patients, which seems to indicate that it is also related to early lung cancer such as GGO. Interestingly, *Lachnospiraceae* can produce anti-inflammatory short chain fatty acids (Louis and Flint, 2009), which seems to be inconsistent with the current result. The difference between studies may be caused by differences in the environment, geographical location, and eating habits. In addition, different sampling methods may also be another reason for different results. Furthermore, it is also related to the heterogeneity of each person’s lung microbiome.

Our results confirm that BALF is indeed vulnerable to contamination by the upper respiratory tract and oral microbiota. In this study, we also used lung tissue directly obtained from surgery, so that we can not only obtain the actual lung tissue microbiome but also reduce possible oral contamination through sample collection. In this study, our results show that the lung microbiota of cancer patients is different from that of other sites of the body, and the most dominant phylum of lung microbiota is *Proteobacteria*, which is also the main phylum of BALF. Compared with BALF samples, there are some different microbiotas of two kinds of samples. The microbiome of lung tissue samples is more complex and the percentage of main microbiota is lower. However, it is worth noting that the main microbiota of the two samples are similar, also the specific proportion is different, this suggests that the microbiome of lung tissue may also be affected by lower respiratory tract microbiota. Our results are partly consistent with previous studies, which revealed the lung microbiome in lung cancer at the phylum level (Mao et al., 2020). However, compared with previous studies, we did not observe the relative abundance difference of *unclassified Comamonadaceae* and *Propionibacterium* at other taxonomic levels between lung cancer and adjacent tissues (Mao et al., 2020), which indicated that there may be differences in the composition of lung microbiome between GGO and typical lung cancer. However, the microbiome characteristics of GGO are still unclear. A small sample study found that the core microbiotas in GGO tissue are *Mycobacterium*, *Corynebacterium*, and *Negativicoccus* (Ren et al., 2019b). Nevertheless, they did not find the different microbiota between GGO and adjacent normal tissues. Our results were partially the same as a recent study, which explored microbiome diversity through tumor tissues of lung ground-glass nodules and solid nodules (Ma et al., 2021). However this study did not involve with the microbiome of BALF, as well as the relationship of microbiome between BALF and tumor tissues. In our study, we found reduced genera including *Ralstonia*, *Lactobacillus*, *unidentified-Chloroplast*, and *Pseudomonas* in the GGO group, Among them, *Ralstonia pickettii* was found to be a mesothelioma specific microbiota involved in tumor progression (Higuchi et al., 2021), and *Lactobacillus* induce anticancer effect by promoting cancer cell apoptosis and preventing oxidative stress, which is common in probiotics (Badgeley et al., 2021), the effect on GGO can mechanically be interpreted by carcinogenesis due to the decreased genera. Interestingly, the most dominant phylum *Proteobacteria* is also significantly reduced. The results indicate that the microbiota in the local microenvironment may also be involved in the initiation and progression of GGO. In our study, all 10 different bacterial genera were used to distinguish GGO and normal lung tissue through the method of random forest analysis, and the AUC was 91.05%, indicating that these bacterial genera have certain value in discriminating GGO and normal lung tissue.

However, our study also has some limitations. First, the sample size is too small to generate credible evidence. Therefore, larger samples and dynamic longitudinal studies are needed in the future to verify the association between microbiome and different pathological types of lung cancer based on different regions and populations. Second, our studies need to combine bacterial and clinical characteristics to raise the ROC value, which indicates that the combined multidimensional data can better predict lung cancer to a certain extent. Finally, we do not obtain lung tissue samples from healthy patients in this study because it is immoral to obtain lung biopsy from healthy subjects, which is also an unsolvable problem for later researchers.

## Conclusion

In conclusion, this is the first time to investigate the microbiome diversity of GGO by BALF combined with lung tissue samples. We found significant differences in the lower respiratory tract and lung microbiome between GGO and the matched non-malignant control group through the BALF and lung tissues. These features may be potential bacterial biomarkers and new targets for GGO diagnosis and treatment.

## Data Availability

The datasets presented in this study can be found in online repositories. The name of the repository and accession number can be found in the following: NCBI; PRJNA843353.
