# Supplementary material for: The microbiome of lower respiratory tract and tumor tissue in lung cancer manifested as radiological ground-glass opacity
Source: Front Bioeng Biotechnol. 2022 Aug 25;10:892613. doi: 10.3389/fbioe.2022.892613 (PMC9455596; doi:10.3389/fbioe.2022.892613)
Supplement: Supplementary file 1 [file DataSheet1.PDF]

Supplementary Material

Zhigang Wu[1†], Jie Tang[1†], Lichen Zhang[1], Chen Gu[1], Ziyue Zhu[1], Runzhou Zhuang[1], Xiao Teng[1], Di Meng[1], Jiacong Liu[1], Jinghua Pang[2], Xiayi Lv[1*]

[1]Department of Thoracic Surgery, The First Affiliated Hospital, School of Medicine, Zhejiang University, Hangzhou, China

[2] Department of Thoracic Surgery, Fenghua People's Hospital, Ningbo, China

[†]These authors contributed equally to this work.

*** Correspondence:**

Xiayi Lv

lyuxiayi@zju.edu.cn

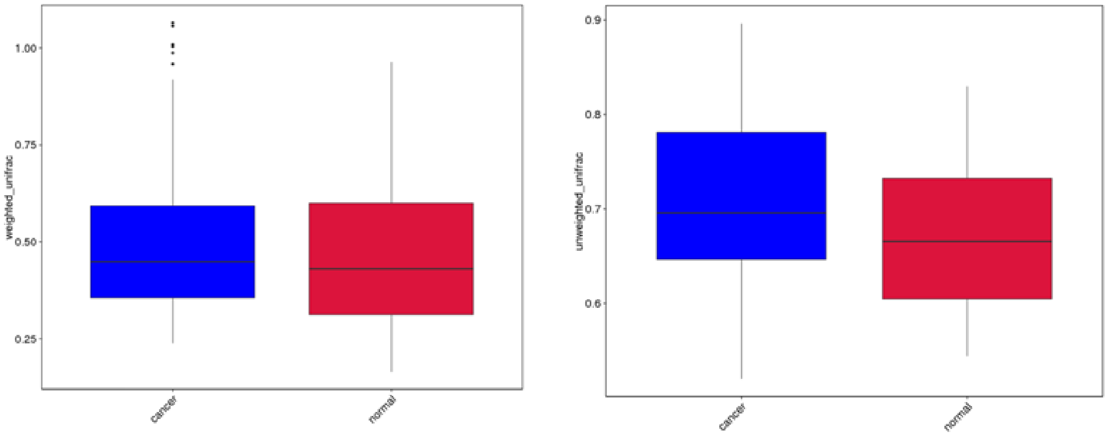

Supplementary Figure 1. Unweighted or weighted UniFrac boxplot of BALF of lung segment with GGO and contralateral normal lung segment

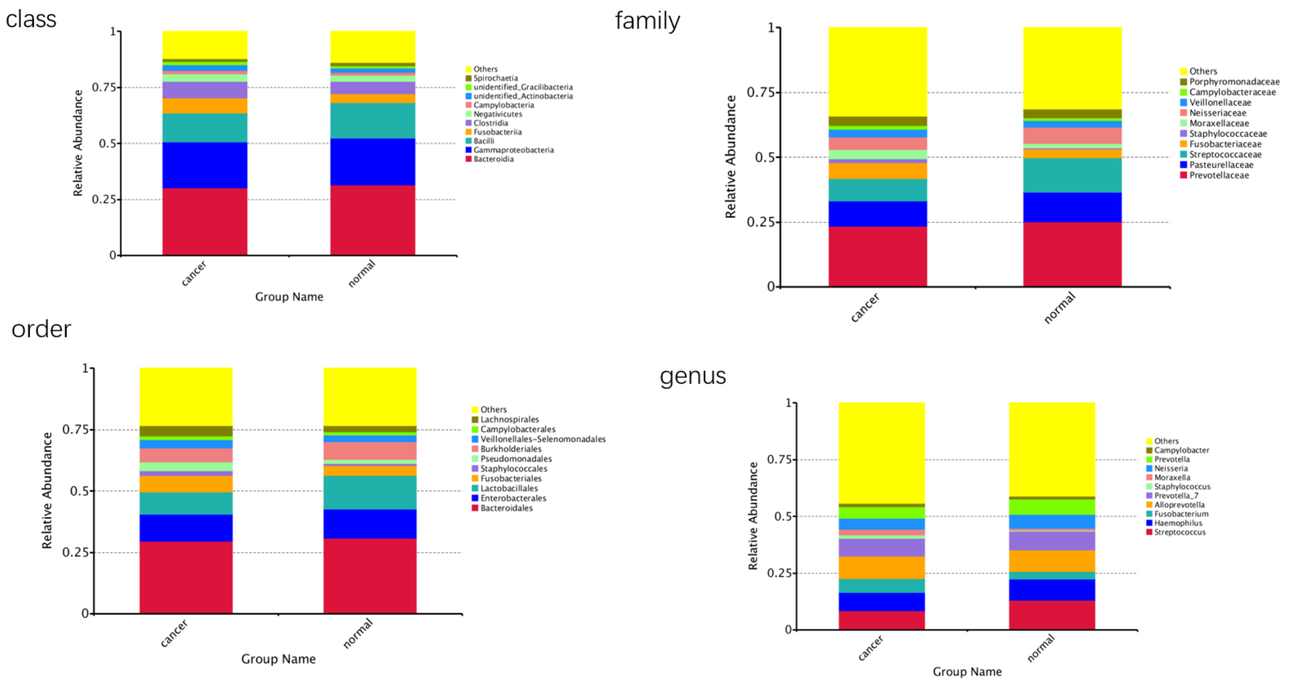

Supplementary Figure 2. The microbiota in the BALF samples of lung segment with GGO and contralateral normal lung segment groups, classification and analysis were based on the class, order, family and genus levels
